# Different humidity environments do not affect the subsequent exercise ability of college football players after aerobic high-intensity interval training

**DOI:** 10.1038/s41598-024-66757-9

**Published:** 2024-07-13

**Authors:** Yongzhao Fan, Ben Zhang, Yan Wang, Hao Wu

**Affiliations:** 1 Department of Physical Education, Henan Normal University, Xinxiang, 453007 Henan China; 2 Department of Arts and Physical Education, Shantou Polytechnic, Shantou, 515078 Guangdong China; 3 Department of Physical Education Teaching and Research, Fuzhou University, Fuzhou, 350108 Fujian China; 4 Capital University of Physical Education and Sports, Beijing, 100191 China

**Keywords:** Physiological responses, Performance, Aerobic high-intensity interval training, Heat and humidity environment, Health policy, Health services, Occupational health, Public health, Quality of life

## Abstract

Previous studies have explored the effect of differing heat and relative humidity (RH) environments on the performance of multiple anaerobic high-intensity interval training (HIIT). Still, its impact on physiological responses and performance following aerobic HIIT has not been well studied. This study examined the effects of differing RH environments on physiological responses and performance in college football players following HIIT. Twelve college football completed HIIT under four different environmental conditions: (1) 25 °C/20% RH (Control group); (2) 35 °C/20% RH (H20 group); (3) 35 °C/40% RH (H40 group); (4) 35 °C/80% RH (H80 group). The heart rate (HR), mean arterial pressure (MAP), lactate, tympanic temperature (T_T_), skin temperature (T_S_), thermal sensation (TS), and rating of perceived exertion (RPE) were recorded continuously throughout the exercise. The heart rate variability (HRV): including root mean squared differences of the standard deviation (RMSSD)、standard deviation differences of the standard deviation (SDNN)、high frequency (HF), low frequency (LF), squat jump height (SJH), cycling time to exhaustion (TTE), and sweat rate (SR) were monitored pre-exercise and post-exercise. The HR, MAP, lactate, T_T,_ T_s,_ TS, and RPE in the 4 groups showed a trend of rapid increase, then decreased gradually. There was no significant difference in HR, MAP, T_T,_ or RPE between the 4 groups at the same time point (p > 0.05), in addition to this, when compared to the C group, the lactate, T_s,_ TS in the other 3 groups significant differences were observed at the corresponding time points (p < 0.05). The RMSSD, SDNN, HF, and LF levels in the 4 groups before exercise were not significantly different. The RMSSD and HF in the H40 and H80 groups were significantly decreased and other HRV indicators showed no significant difference after exercise. In sports performance measurement, the SJH and TTE were significantly decreased, but there was no significant difference in the 4 groups. The SR was no significant difference in the 4 groups after exercise. In conclusion, heat and humidity environments elicited generally greater physiological effects compared with the normal environment but did not affect sports performance in college football players.

## Introduction

High-intensity interval training (HIIT) is a renowned, and time-efficient, method of training that can improve athletes' cardiorespiratory and metabolic function, which in turn improves their physical performance^1,2^. It is worth mentioning that preceding studies have explored heat exposure on the performance and recovery from multiple anaerobic HIIT bouts^3^. However, it has been confirmed in previous studies that aerobic 4 × 4-min HIIT is significantly more effective than performing the same total work at either lactate threshold or 70% of maximum heart rate, in improving maximum oxygen uptake^4^. Meanwhile, according to the literature, the 4 × 4 min interval running is also commonly applied in the daily training of athletes. For instance, a previous study showed that an elite national cyclist improved maximal oxygen uptake and time trial performance in the preseason period by reducing total training volume and increasing 4 × 4 min HIIT^5^. Besides, numerous studies have reported on the application of 4 × 4 min HIIT in football training. Early research found that the maximal oxygen uptake, lactate threshold, running economy, the number of sprints, and involvement with the ball were increasing during a football match in the male elite junior football players after 8 weeks of 4 × 4 min HIIT^6^. Then, several papers likewise reported similar findings in the field of football training^7,8^. Up to now, the small-sided game (SSG) is an adapted form of the game, often used in football training^9^. Typically, SSG is prescribed similarly to those recommended for HIIT^10^.

Moreover, football is played in many different environments, and competitive matches where temperatures can exceed 30 degrees Celsius and relative humidity (RH) is high^11^. Consequently, football players must live, train, and play in high temperatures and with varying humidity^12^. Hot conditions have a significant influence on the physiology and aerobic performance of athletes^13^. Furthermore, previous studies suggested that the combination of hot conditions and elevated relative humidity would bring substantial stress to athletes^14^. This is because the evaporative capacity of the environment decreases when the humidity rises at high temperatures^15^. However, early studies in this field concentrated on exploring the influence of high temperature and humidity environments on the maximum oxygen uptake, the Wingate anaerobic test performances, and prolonged exercise capacity^16,17^. In addition, some studies explored the impact of differing humidity conditions on low-intensity and prolonged exercise in a hot^18^ and warm environment^19^, respectively. However, whether humidity changes in a hot environment affect the physiological responses and performance of football players following 4 × 4 min HIIT has not been well studied.

Hence, the purpose of our study was to investigate the physiological responses and performance of college football players following HIIT in heat and different humidity environments. Our research hypothesis was that high temperature and different humidity environments significantly affected the physiological performance and athletic ability of college football players following 4 × 4 min HIIT.

## Methods

### Research design

**Figure 1:**
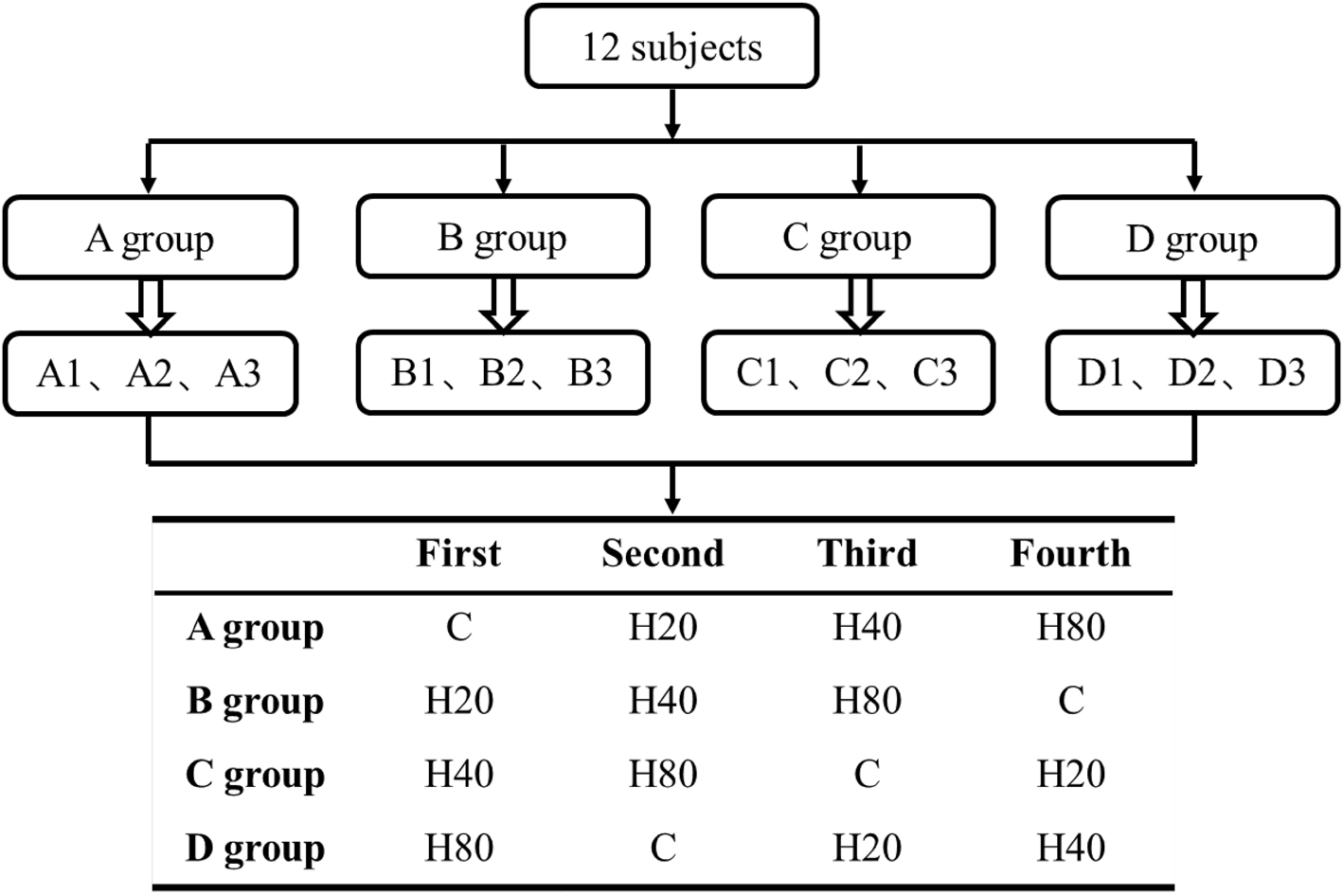
Sample cross-over experiment table.

In this study, a randomized controlled, cross-over experimental design was applied. Subjects were numbered and randomly divided into 4 groups of 3 each by the random function method in Excel. Then, the experiment was conducted according to the contents of the cross-tab in Fig. 1 by entering the corresponding environment in turn. The experimental environment was regulated by a combination of heating (SAWO, CON4, Finland) and humidification (BELIN, SC-G060ZS, CHN) equipment and all experimental conditions could be completed according to the experimental settings with an ambient and humidity error range of ± 1 °C. Subjects entered the laboratory one week before the start of the experiment to familiarize the procedures and equipment, and four days before the start of the experiment to take body morphometry (DXA, Lunar Prodigy, GE, USA) and perform athletic ability tests.

On the day of the test, subjects underwent the same warm-up protocol^20^ after the basal index collection and then completed the experiment according to the prearranged schedule. Basal indicators (BL) collected were heart rate (HR), mean arterial pressure (MAP), lactate, tympanic temperature (T_T_), skin temperature (T_S_), thermal sensation (TS), and rating of perceived exertion (RPE), heart rate variability (HRV), and weight. HR, lactate, T_T_, Ts, TS, and RPE are monitored immediately or 3 min after exercise^21^ and every 5 or 10 min^22^ for 50 min after exercise. HRV was taken at 55 min and SJ, and TTE were collected at the 60th minute post-exercise. The experiment conducted between December 2020 and April 2021 and not be affected by the natural environment. To avoid the effect of circadian rhythms on this experiment, all subjects experimented at the same time for each test. In addition, subjects were guaranteed a healthy proportion of food intake under the condition that food intake remained constant. There were two dedicated experimenters in the laboratory, one collected data and the other verified and record the collected data.

### Subjects

**Figure 2:**
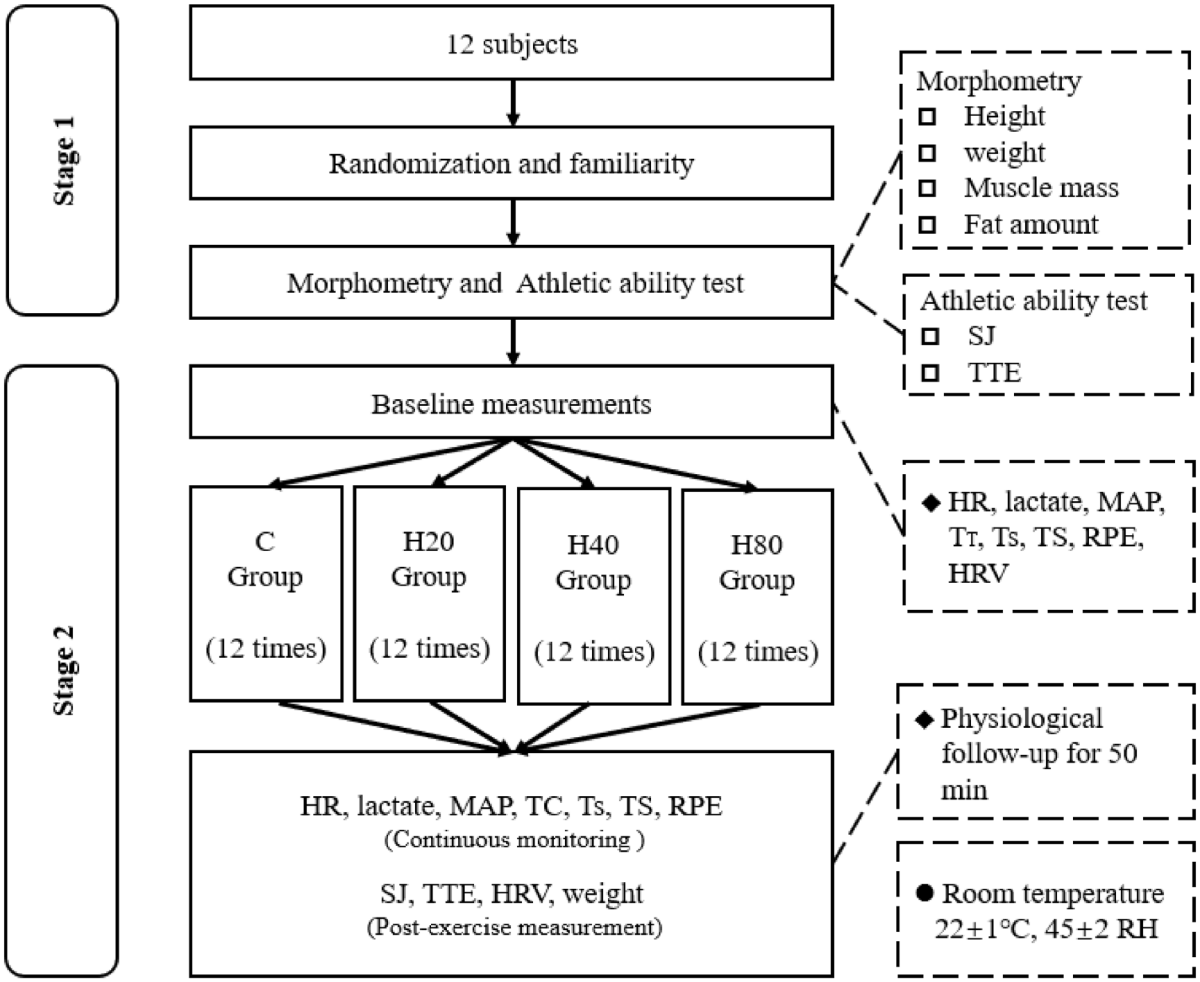
Overall experiments timeline.

G*Power software (Version No.3.1.9.7. Franz Faul University Kiel, Germany) was used to calculate the sample size for this experiment^23^. The specific parameters were set as follows: α = 0.05, power = 0.80, effect size = 0.25, statistical test = repeated measures, with-between interaction, number of groups = 4, number of measurements = 2. Under the above conditions, the resulting data size was 9 subjects per group. However, 12 subjects were selected for this study to prevent an insufficient amount of data following sample attrition. The basic information of the twelve college football players is shown in Table 1. Subjects completed HIIT in four different environmental conditions, respectively: ① 25 °C/20% RH; ② 35 °C/20% RH; ③ 35 °C/40% RH; ④ 35 °C/80% RH. The elution period between each experiment was 1 week. All subjects were free of high-intensity exercise for 3 days before the experiment. In addition, the subjects were free from undesirable habits and did not consume tea, functional drinks, or nutritional supplements during the conduct of the experiment. The informed consent was obtained from all subjects. The institutional ethical committee of the Capital University of Physical Education and Sports, Beijing, China approved all procedures and protocols (No. 2021A43). The study was following the Declaration of Helsinki. An overall experiment timeline is shown in Fig. 2.

**Table 1:** Basic subject information.

Number	Age	Height (cm)	Weight (kg)	Muscle mass (kg)	Fat rate (%)	Training years	VO^2^max (ml/kg/min)
12	25.09 ± 1.65	176.96 ± 1.51	71.55 ± 4.59	56.14 ± 4.19	12.06 ± 1.48	6.75 ± 1.14	46 ± 5.1

### Exercise protocol

**Figure 3:**
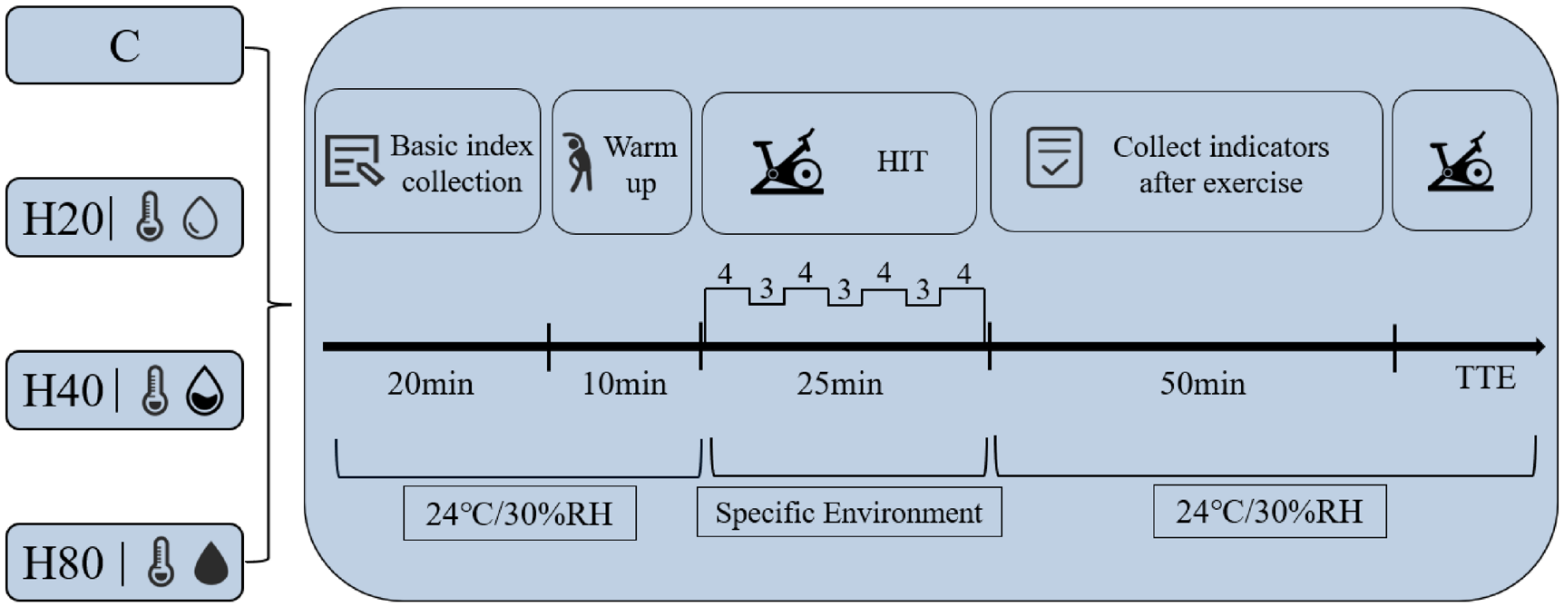
Detailed experimental procedure.

The exercise protocol was 4 × 4 min HIIT: 4 × 4-min intervals at 90–95% HRmax with 3 min of active rest between each interval at 70%HRmax^4^. Due to the limitations of the experimental environment, the exercise protocols were completed on a cycle ergometer (Aerobike 75XL, Combi, Tokyo, Japan). Subjects maintained the seat height of the cycle ergometer at the same height as their greater trochanter height when standing throughout the experiment. The detailed experimental procedure is shown in Fig. 3.

### Physiological indicators measurement

#### Heart rate

The subject wore an HR monitor (Polar H10, Finland) upon entering the laboratory and then sat still for 10 min to obtain the basal HR. Then, after a warm-up exercise, the subject entered the specific environment. The HR was monitored during the completion of the pre-determined exercise protocol and was taken immediately after exercise and every 5 min after exercise until the 50th minute.

#### Mean arterial pressure

Similar to heart rate, the diastolic blood pressure and systolic blood pressure of the subject were collected using an electronic sphygmomanometer (YE660A, YUWELL, CHN) worn on his or her left arm. The MAP was obtained by the following formula: MAP = diastolic blood pressure + (systolic blood pressure − diastolic blood pressure)/3^24^

#### Lactate

Lactate was obtained by testing ear blood with a blood lactate meter (Lactate Scout, Germany). Lactate collection was taken 3 min immediately after exercise^25^ and every 5 min after exercise until the 50th minute.

#### Sweat rate

The SR was calculated by collecting information on the body weight of the subject before and after exercise and using the following formula: Sweat rate = (Δmass + fluid—urine – blood)/exercise time^20^.

### Temperature measurement

#### Tympanic temperature

T_T_ was achieved by ear thermometer (OMRON TH839S, CHN) and this method was a non-invasive method. Furthermore, to obtain an accurate T_T_, the T_T_ test was measured by a specific person, the depth of measurement was kept consistent for each subject throughout the experiment and the ear thermometer was cleaned and the measuring area laminate was changed regularly^26^.

#### Skin temperature

The T_S_ (OMRON MC-872, CHN) was obtained by testing the subject's temperature at 4 locations: neck (T_neck_), right infraspinous fossa (T_scapula)_, right dorsal hand (T_hand)_, right mid-shin (Tshin) and calculated using the following formula: Mean T_S_ = (T_neck_ × 0.28) + (T_scapula_ × 0.28) + (T_hand_ × 0.16) + (T_shin_ × 0.28)^27^. Similarly, to avoid errors due to human factors, this operation was carried out by a special person.

### Autonomic function measurement

#### HRV measurement

This experiment was chosen to evaluate the effects of different environments on autonomic function by testing changes in HRV before and after exercise in subjects. When collecting HRV indicators, the subject was placed in a quiet, softly lit room for 4 min while wearing the Omegawave autonomic assessment sensor (Omegewave, Personal version, Finland)^28^ and was not allowed to move any part of their body or speak during the test. The experiment focused on the time (RMSDD, SDNN) and frequency domains (HF, LF) of HRV.

### Subjective indicator measurements

#### Thermal sensation

A nine-point standard heat sensation scale was used in this study, where subjects were asked to state they had true heat sensations at the corresponding time points before and after exercise, according to the scale^29^.

#### Rating of perceived exertion

A twenty-point standard perceived exertion scale was used in this study, where subjects were asked to state they truly perceived exertion at the corresponding time points before and after exercise, according to the scale^30^.

### Sport performance measurement

#### Squat jump height

The SJH was obtained by applying a vertical jump pad (Omegewave, Finland). At 55 min post-exercise, the participant stood on a vertical jump pad with feet shoulder-width apart, then placed hands on the iliac bones, squats down to 90°, rested for 2 s, then jumped as hard as he could to the highest point and finally dropped to the mat. This movement was tested 3 times and took the average (ASJ) and peak squat jump height (PSJ)^31^.

#### Time to exhaustion (TTE)

The TTE was often used as a measure of endurance performance in a laboratory environment^32^. The subject started the cycling (LODE 906900, The Netherlands) at 50 W and increased 50 W every 2 min until exhaustion. The cycling time was recorded. Two criteria for determining subject exhaustion: (1) the subject has reached or exceeded his maximum heart rate; (2) the subject was unable to cycle at the specified intensity, which was defined as a required drop in cycling cadence of 10 rpm/min for a duration of greater than 20 s^33^.

### Statistics and analysis

Descriptive results were reported as means ± standard deviations (SD). The assumption of normality was verified using the Shapiro–Wilk test. The physiological indicators except for sweat rate (SR) indicators were analyzed using repeated measures ANOVAs mixed design with environments (C, H20, H40, and H80) as between factor, and time (for physiological measurements: baseline, 0, 3, 5, 10, 15, 20, 25, 30, 35, 40, 45, 50 min as within factor. Post-hoc analyses using Bonferroni correction were performed where appropriate. SR was analyzed using one-way ANOVAs with Bonferroni correction. All statistical analyses were performed using the statistical package SPSS 25.0, with the level of significance at P < 0.05.

## Results

### Physiological indicators measurement

#### Heart rate

**Figure 4:**
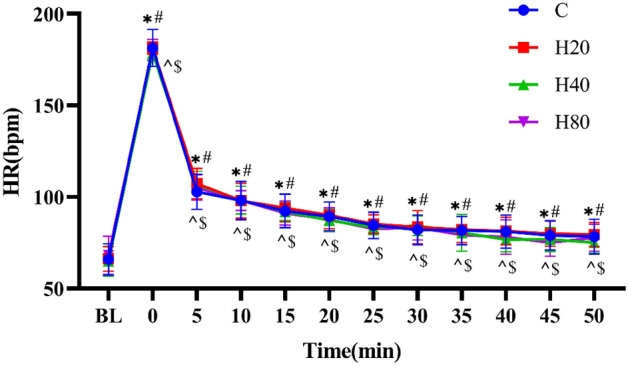
The dynamic changes in HR in 4 groups over 90 min. *p < 0.001, ^#^p < 0.001, ^^^p < 0.001, ^$^p < 0.001 compared to baseline in C, H20, H40 and H80 group, respectively.

As shown in Fig. 4, the HR of the 4 groups was significantly increased immediately (p < 0.001) and even 50 min after exercise (P < 0.01) when compared with pre-exercise. No differences were detected in HR between the 4 groups at the same time point (p > 0.05).

#### Mean arterial pressure

**Figure 5:**
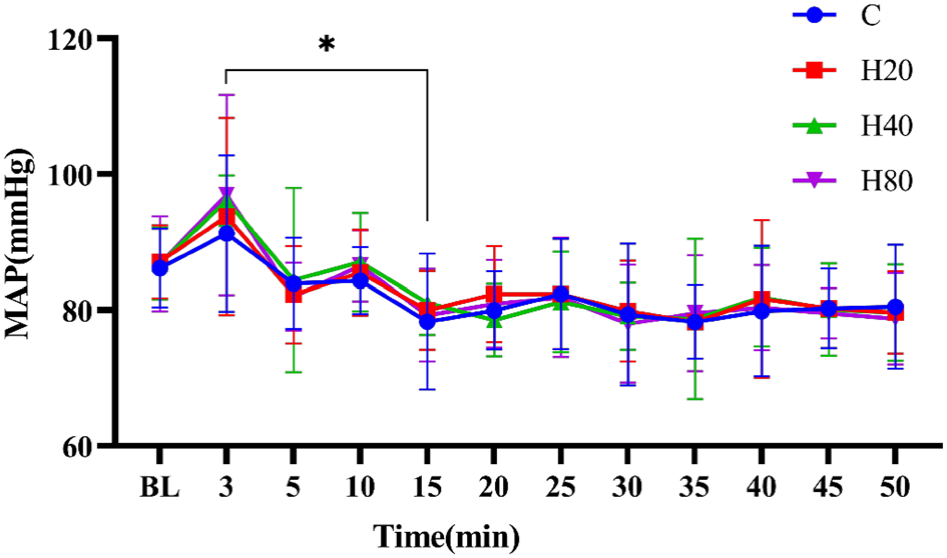
The dynamic changes in MAP in 4 groups over 90 min. *p < 0.05, 3 min vs 15 min in 4 groups.

As shown in Fig. 5, the MAP of the 4 groups was increased at 3 min (p > 0.05) when compared with pre-exercise. Besides, MAP was significantly lower at 15 min (p < 0.05) compared with 3 min after exercise. No differences were detected in HR between the 4 groups at the same time point.

#### Lactate

**Figure 6:**
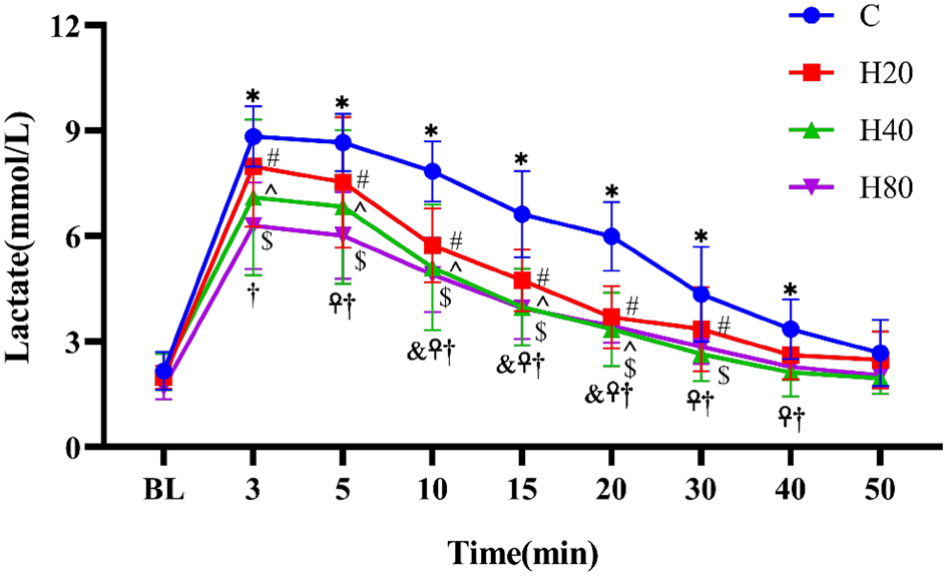
The dynamic changes in lactate in 4 groups over 90 min. The *p < 0.001, ^#^p < 0.001, ^^^p < 0.001 and ^$^p < 0.001 compared to the baseline in the C, H20, H40, and H80 group, respectively. ^&^p < 0.01, C vs H20. ^☥^p < 0.05, C vs H40. ^†^p < 0.01, C vs H80.

As shown in Fig. 6, when compared with the baseline level, the lactate in the C group after exercise showed a trend of rapid increase, reached the maximum value (P < 0.001) at 3 min, and then decreased gradually, the lactate levels are still greater than basal at 40 min after exercise. The lactate levels of rats in the H20, H40, and H80 groups showed the trend was similar to the C group, and also reached maximum value (P < 0.001) at 3 min, but the increasing magnitude of rats in H20, H40, and H80 group was smaller (P < 0.05). When compared with the C group at the same time point, H20, H40, and H80 groups were smaller. No differences were detected in lactate between the H20, H40, and H80 groups at the same time point.

#### Sweat rate

**Figure 7:**
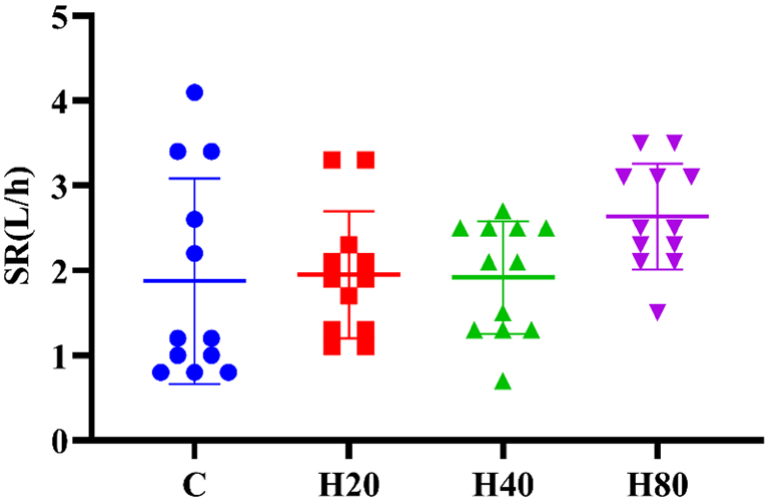
The SR of each group after exercise.

As shown in Fig. 7, the SR was no significant difference in the 4 groups [F_(3,44)_ = 2.191, P = 0.103]. Although the SR of the H80 group was increased, there was no significant difference when compared with the C, H20, and H40 groups (P > 0.05).

### Temperature measurement

#### Tympanic temperature

**Figure 8:**
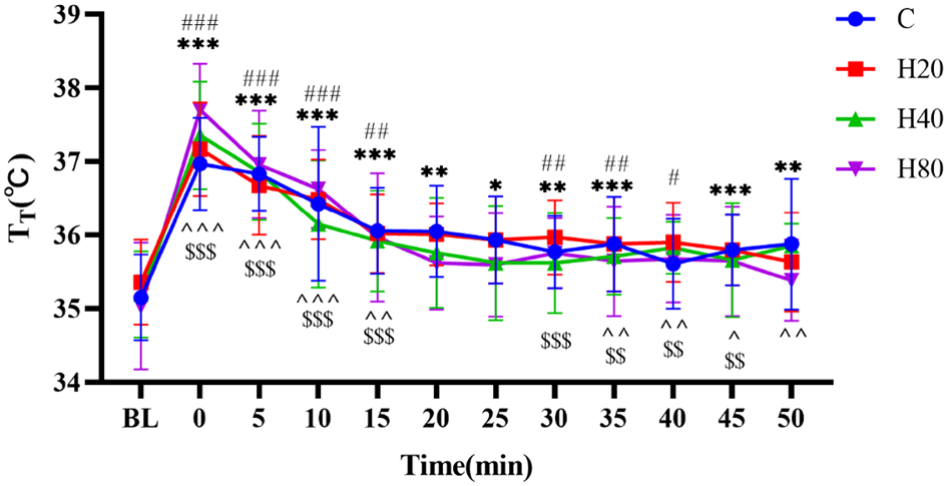
The dynamic changes in T_T_ in 4 groups over 90 min. The ***p < 0.001, **p < 0.01, *p < 0.05 compared to baseline in C group, ^###^p < 0.001, ^##^p < 0.01, ^#^p < 0.01 compared to baseline in H20 group, ^^^^^p < 0.001, ^^^^p < 0.01, ^^^p < 0.05, compared to baseline in H40 group, ^$$$^p < 0.001, ^$$^p < 0.01, ^$^p < 0.05 compared to baseline in H80 group.

As shown in Fig. 8, when compared with the baseline level (C35.15 ± 0.58), the T_T_ in the C group after exercise showed a trend of rapid increase, reaching maximum value immediately after exercise, and then decreasing gradually. The T_T_ was still greater than the basal at 50 min after exercise. The T_T_ in H20, H40, and H80 groups showed the trend was similar to the C group, and also reached the maximum value (P < 0.001) immediately after exercise, but the increasing magnitude of H20, H40, and H80 groups were smaller (P < 0.05). When compared with the C group at the same time point, H20, H40, and H80 groups were smaller. No differences were detected in T_T_ between the H20, H40, and H80 groups at the same time point.

#### Skin temperature

**Figure 9:**
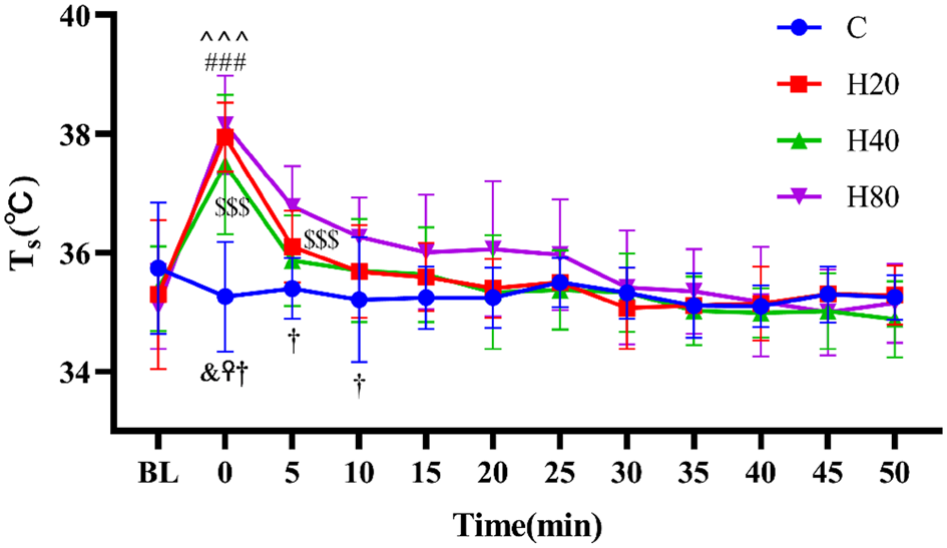
The dynamic changes in T_S_ in 4 groups over 90 min. The ^###^p < 0.001, compared to baseline in H20 group, ^^^^^p < 0.001, ^^^^p < 0.01, compared to baseline in H40 group, ^$$$^p < 0.001, compared to baseline in H80 group. ^&^p < 0.01, C vs H20. ^☥^p < 0.01, C vs H40. ^†^p < 0.01, C vs H80.

As shown in Fig. 9, the T_S_ in the C group remained flat before and after exercise (P > 0.05). When comparing with baseline level, the T_S_ in H20, H40, and H80 groups after exercise showed a trend of rapid increase, reached maximum value immediately after exercise (P < 0.001), and then decreased gradually, the T_S_ was still greater than basal at 5 min in H80 group, separately. When compared with the C group, the H20, H40, and H80 groups were higher immediately after exercise, and the H80 group was also higher at 5 and 10 min. No differences were detected in T_S_ between the H20, H40, and H80 groups at the same time point.

### Autonomic function measurement

#### Heart rate variability

**Figure 10:**
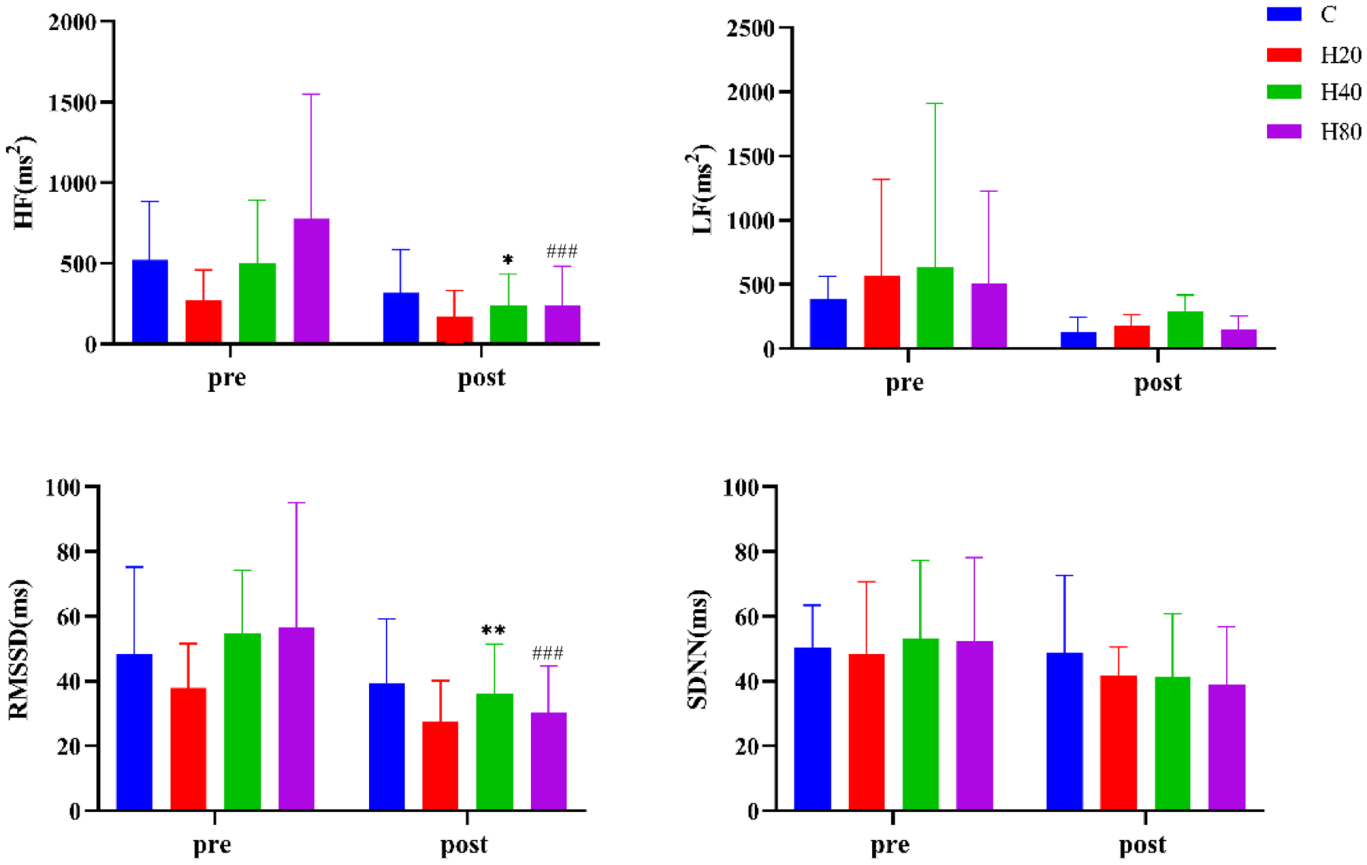
The dynamic changes in HRV in 4 groups. The *p < 0.05, **p < 0.01, compared pre-exercise with post-exercise in H40 group, respectively. ^###^p < 0.001 compared pre-exercise with post-exercise in H80 group.

As shown in Fig. 10, the HF, LF, RMSSD, SDNN levels in the 4 groups before exercise were not significantly different. The HF in the H40 and H80 groups were significantly decreased and other HRV indicators showed no significant difference after exercise.

### Subjective indicator measurements

#### Thermal sensation

**Figure 11:**
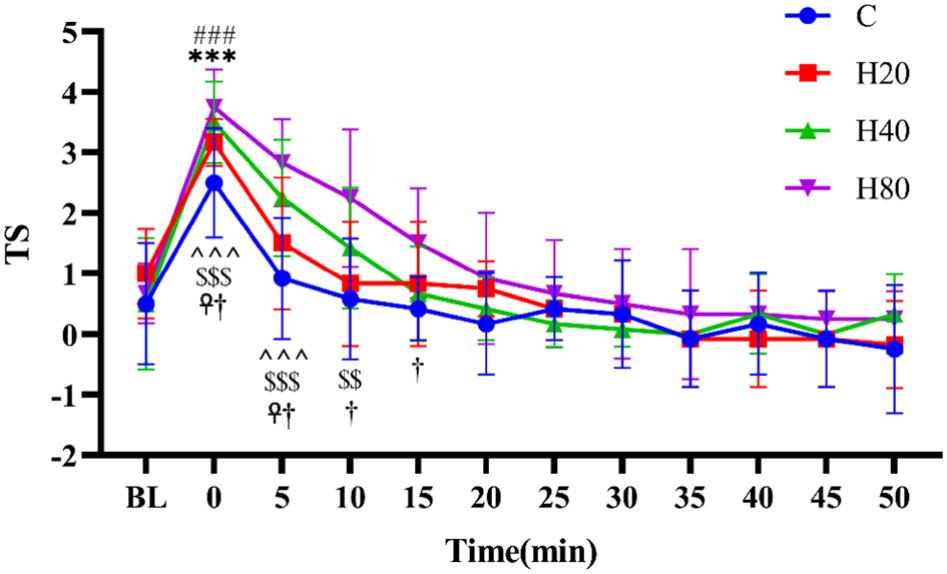
The dynamic changes in TS in 4 groups over 75 min. The ***p < 0.001, ^###^p < 0.001, and ^^^^^p < 0.001 compared to the baseline in the C, H20, and H40 group, respectively. ^$$$^p < 0.001, ^$$^p < 0.01, compared to baseline in H80 group. ^☥^p < 0.01, C vs H40. ^†^p < 0.01, C vs H80.

As shown in Fig. 11, when compared with the baseline level, the TS in the C group after exercise showed a trend of rapid increase, reached maximum value immediately after exercise, and then decreased gradually. The TS in the H20, H40, and H80 groups showed the trend was similar to the C group, and also reached maximum value immediately after exercise, but the increasing magnitude of the H20, H40, and H80 groups were larger (P < 0.05). When compared with the C group, the TS in the H40 and H80 groups were larger at 0 and 5 min, and the TS in the H80 groups were also larger at 10 and 15 min. No differences were detected in TS between the H20, H40, and H80 groups at the same time point.

#### Rating of perceived exertion

**Figure 12:**
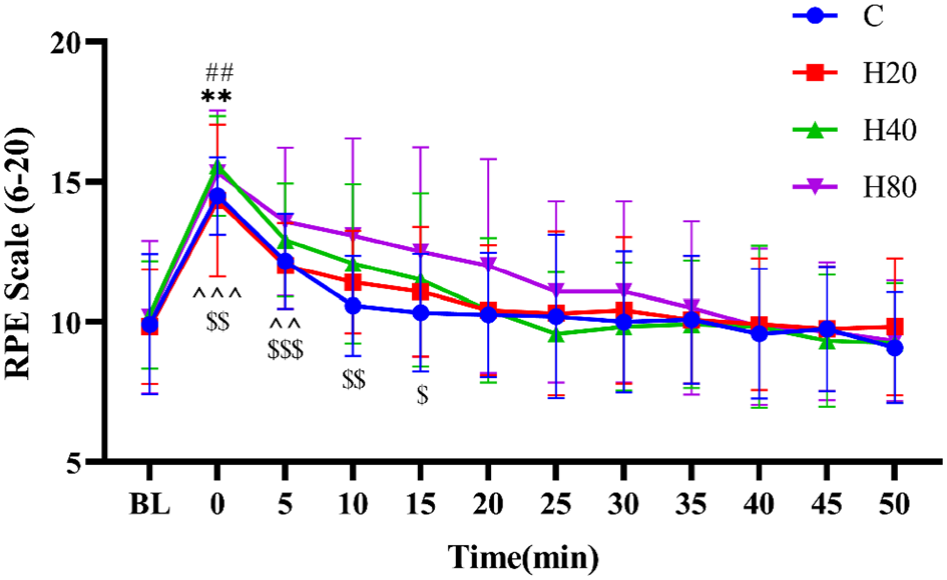
The dynamic changes in RPE in 4 groups over 75 min. The **p < 0.01, ^##^p < 0.01, ^^^p < 0.001compared to baseline in the C, H20 and H40 group, respectively. ^$$$^p < 0.001, ^$$^p < 0.01, ^$^p < 0.05 compared to baseline in H80 group.

As shown in Fig. 12, when compared with the baseline level, the RPE in the C group after exercise showed a trend of rapid increase, reaching maximum value immediately after exercise (P < 0.01), and then decreasing gradually. The RPE in H20, H40, and H80 groups showed the trend was similar to the C group, and also reached maximum value immediately after exercise (P < 0.01). In addition, when compared with the baseline level, the RPE in the H40 and H80 groups were higher at 5 min and the RPE in the H80 group was also higher at 10 and 15 min. No differences were detected in RPE between the 4 groups at the same time point.

### Sport performance measurement

#### Squat jump height

**Figure 13:**
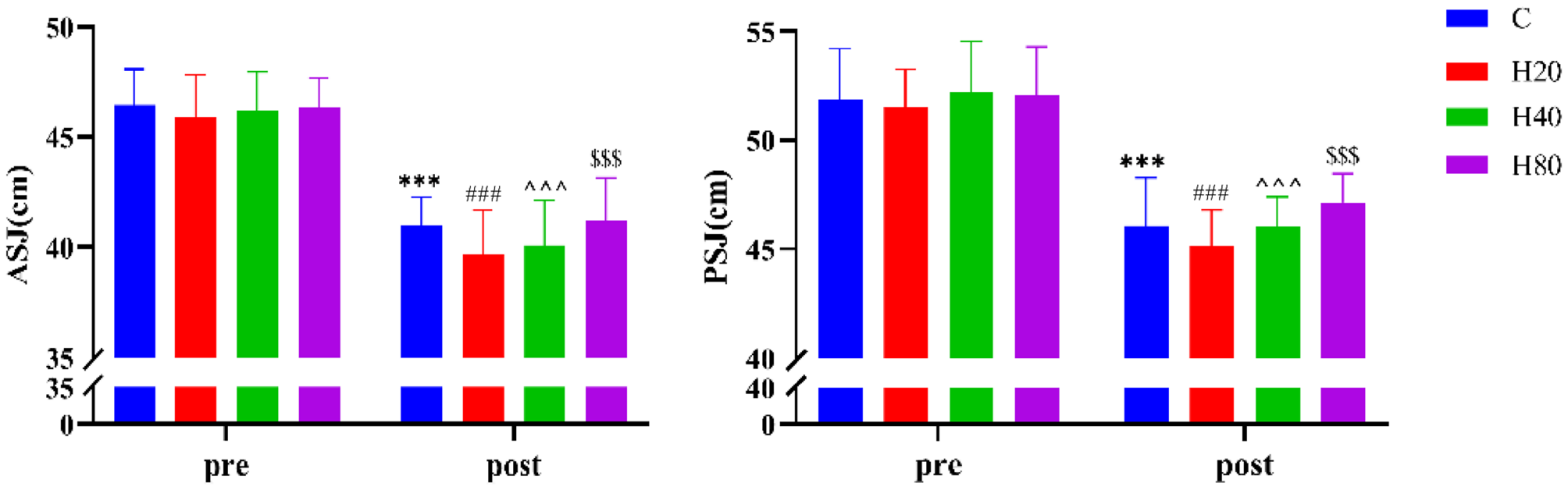
The dynamic changes in SJH in 4 groups. The ***p < 0.001, ^###^p < 0.001, ^^^p < 0.001, ^$$$^p < 0.001 compared pre-exercise with post-exercise in the C, H20, H40, and H80group, respectively.

As shown in Fig. 13, the ASJ and PSJ in the 4 groups before the exercise showed no significant difference. The ASJ and PSJ in the 4 groups were significantly decreased and there was no significant difference between the 4 groups after exercise.

#### Time to exhaustion (TTE)

**Figure 14:**
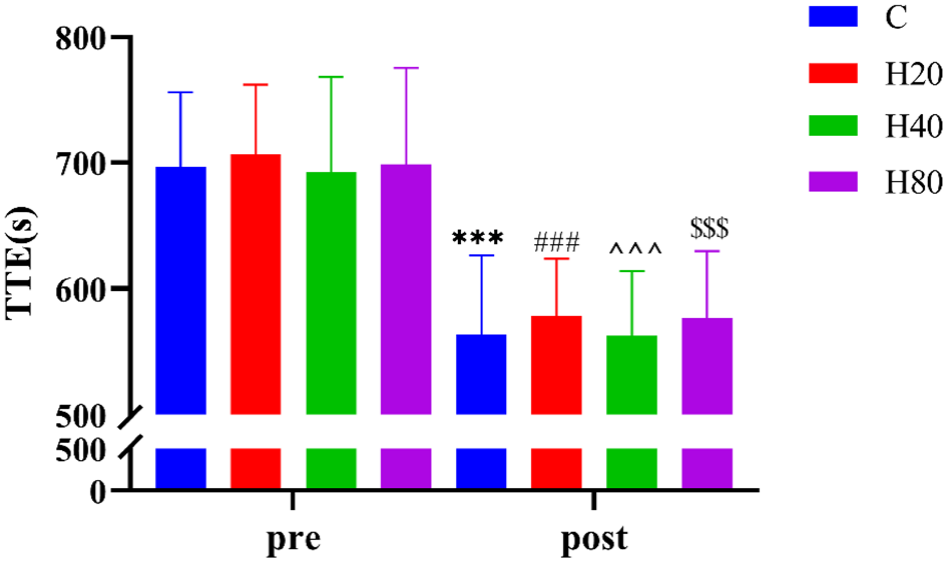
The dynamic changes in TTE in 4 groups. The ***p < 0.001, ^###^p < 0.001, ^^^p < 0.001, ^$$$^p < 0.001 compared pre-exercise with post-exercise in the C, H20, H40, and H80group, respectively.

As shown in Fig. 14, the TTE in the 4 groups before exercise showed no significant difference. The TTE in the 4 groups was significantly decreased and there was no significant difference between 4 groups after exercise.

## Discussion

It is well known that high-temperature environments can have significant effects on the cardiovascular system and exercise capacity of athletes^34,35^, however, the effects of high-temperature environments with different humidity conditions on athletes have been less studied, especially those with high-intensity intervals as the exercise mode have not been well investigated^19,36^, therefore, this study was conducted to investigate this issue.

The HIIT for football players consisted of four times 4 min at 90–95% of maximal heart rate, with a 3-min jog at 70% of maximal heart rate in between^37^. The average age of the college football players in this experiment was 25 years old. The average heart rate during the HIIT protocol was about 180 beats, therefore, the execution of the exercise protocol in this experiment was completely reliable. In addition, because in practice and this experiment, the monitoring of actual HR was mainly used as the basis for exercise intensity^7^, the main exercise modality used in this study was the cycle ergometer. On the one hand, studies have shown that the differences in cardiovascular responses to treadmill and cycle ergometer exercise in children and adults were not exercise modality dependent^38^. On the other hand, in high temperature and humidity environments, especially under high humidity conditions, water in the air is prone to condense on the treadmill, causing safety hazards and cycle ergometer is safer^39^. However, it is worth mentioning that due to the 4 × 4 HIIT exercise modes of football players are different compared to that on the cycle ergometer, it is recommended to test it in a real environment.

In addition, the HR of the 4 groups was significantly increased immediately and even 50 min after the completion of the HIIT protocol when compared with pre-exercise. Because using an exercise program with HR as the intensity, no differences were detected in HR between the 4 groups immediately after exercise and during the recovery process, in addition, the HR at the corresponding time point after exercise was still higher than the baseline level among the four groups. The results of this study are consistent with those of previous studies. One study showed that the HR of male amateur football players was significantly higher than rest at the 5 min underwent a single bout of supramaximal exercise^24^. Another study showed that the HR in a subject undergoing 30 min of moderate dynamic exercise could not reach the baseline followed by a 30-min recovery period^40^. Our study also found that in a hot and humid environment, football players had a significantly higher HR in the 20th minute after a high-intensity interval exercise than before the exercise^41^. Numerous studies have proven that after exercise, the sharp drop-in heart rate does not reach its previous resting value, but continues to fall slowly in an exponential manner, depending on the duration and intensity of the exercise^42–44^. According to ACSM recommendations, 90% HRmax is equal to 85% of maximal oxygen uptake^45^, and our study was a long period of high-intensity exercise, so the above phenomenon was reasonable after exercise. Furthermore, a study similar to our study found that 60 min after HIIT in differing heat and humidity exercises, the heart rate was significantly higher than the resting heart rate and this was not significantly different between the groups^46^.

Exercise led to a marked change in cardiac autonomic balance^47^. Using a combination of time- and frequency-domain methods, it has been established in various studies that at the beginning of exercise, cardiac vagal tone immediately decreased and increased the effects of cardiac sympathetic activation as the HR continued to rise^48^. HR dropped rapidly when high-intensity exercise was discontinued. Reactivation of cardiac parasympathetic nerves was the main factor that determines the immediate decrease in HR when exercise was stopped or intensity was decreased^49,50^.

Moreover, our study found that football players in 4 groups showed a decrease in both times- and frequency-domain metrics and did not return to baseline at the 50th minute after the HIIT exercise. Consistent with previous studies, it has been shown that during exercise, both time and frequency domain variables decrease compared to resting values^51^, RMSSD and HF converge to zero^52^, and then HRV values begin to recover immediately after exercise but do not reach baseline levels for 10 min^53^ or 60 min after exercise^54^. Similarly, one study analyzed the changes in HRV during/after exercise in hot and humid environments, the RMSSD and HF were significantly decreased during exercise, did not return to basal levels until the 8th hour after exercise and there is no significant difference between different environments^55^.

A similar trend to HRV, the MAP increased significantly immediately after exercise, decreased rapidly after exercise, and was significantly lower than the maximum value in the 15th minute after exercise, and was less than the basal level within 50 min after exercise, although there was no significant difference compared to the basal value. Consistent with the results of the present study, a previous study found that systolic blood pressure in subjects rose to its highest value in the third minute after exercise and was significantly lower than basal values in the 30th minute after exercise^56^. Another study confirmed that MAP was significantly reduced to pre-exercise levels at the 60th minute after exercise^57^. Indeed, the hypotension generated following HIIT exercise was the result of sympathetic-vagal rebalancing^58^. On the other hand, our study also revealed no significant difference in MAP between the 4 groups after exercise under different temperature and humidity conditions. Early studies found that changes in humidity within the environment did not cause changes in blood pressure^59^. However, one study confirmed that a hot environment significantly increased MAP after exercise in untrained men^60^. We believe that the reason for the discrepancy between the results of this study and ours may be due to differences in the exercise classes of the participants studied, it has been shown that compared to untrained individuals, well-trained athletes have a faster percentage decrease of the blood pressure after exercise^61^.

The lactate in 4 groups after exercise showed a trend of rapid increase, reached a maximum value at 3 min, and then decreased gradually, the lactate levels are still greater than basal at 40 min. Consistent with the results of the present study, earlier studies found that lactate levels in subjects did not return to basal values until the 50th minute after completion of a 5-min high-intensity training session^62^. Another study confirmed that lactate levels were significantly higher than basal values in trainers and untrained men at the 20th minute after completing 3 sets of supramaximal exercise^63^. A recent study, also consistent with our results, found that 15 min after completing high-intensity functional training, the lactate levels of athletes remained at high levels^64^. Moreover, our study also found the increasing magnitude of lactate levels in the H20, H40, and H80 groups became progressively smaller with increasing temperature and humidity. This may be the result of our study protocol, which uses heart rate as a measure of exercise intensity, but numerous studies have confirmed that increases in temperature and humidity cause an increase in HR^19,65–67^. The HR would remain higher in a hot and humid environment, allowing the subjects to achieve a higher HR with relatively low resistance, while lactate is an indicator of exercise intensity^68^, thus causing a decrease in lactate levels at the same HR.

Immediately after the HIIT exercise, T_T_ increased significantly in all 4 groups, and the higher the ambient temperature and humidity, the higher the core temperature. Ts also increased significantly in H20, H40, and H80 groups. Previous research has demonstrated that systematically increasing humidity in a hot environment can lead to increasing T_T_ and Ts in trained distance runners^17^. The reason for this is that in the resting state, radiation, convection, and evaporation are usually the three main pathways for heat dissipation. During exercise, evaporation is the main pathway since radiation and convection cannot increase much from resting values. However, as the humidity rises, the metabolic thermal efficiency decreases, which is the main reason for the increase in T_T_ and Ts^69,70^. In addition, our study also observed that T_T_ remained significantly higher than basal values at the 35th minute after exercise even under normal temperature conditions. Similar results were reported in a previous study, which found that the core temperature of male endurance-trained cyclists remained high at 60 min after completing an all-out 35-min exercise^71^. Furthermore, our study also found that performing HIIT under normal temperature and humidity conditions did not cause changes in Ts, which is also consistent with previous studies. Early studies have found that even when athletes are exposed to high temperatures prolonged endurance exercise did not cause significant changes in Ts during and after exercise^72^.

In the sports performance test, we chose two kinds of tests: squat jump and TTE. The results of the study found that a significant decrease in ASJ, PSJ, and TTE occurred in 4 groups. Consistent with our study, a previous study found that the vertical jump height of football players was significantly decreased within 48 h after completing a one-off football match^73^. Another study confirmed that TTE was significantly reduced when performing a second cycling time to failure experiment in normothermia^74^. The above results are caused by a decrease in muscle contraction strength^75,76^. The underlying causes may be impaired excitation–contraction coupling^77^ and less calcium is released per action potential^78^. As a result, there was no ability to activate the complete force-generating structure^79^. The reduction in the availability of calcium ions to the myocyte will resulted in a decrease in the strength of myocyte activity and thus in the strength of muscle contraction^80^. Moreover, our study found there was no significant difference between the 4 groups in the subsequent sports performance test, the above reasons may be the result of adopting HR as an indicator of intensity as we mentioned above.

In subjective indicator measurements, we collected TS and RPE indices from subjects under different temperature and humidity conditions and found that TS indices were significantly increased after HIIT exercise in 4 groups, when compared with the C group, the TS in H40 and H80 groups were larger at 0 and 5 min, and the TS in H80 groups were also larger at 10 and 15 min. During exercise, about 98% of heat dissipation from the body is through sweat evaporation^81^. However, as the temperature and relative humidity in the environment increase, it will make it difficult for sweat to evaporate, thus increasing the body temperature and thus increasing the heat sensation value^82^. Additionally, our study found that the RPE demonstrated a similar change in trend to TS. Unlike TS, the RPE of the 4 groups was not significantly different. Only the H80 group remained above the basal value at 15 min post-exercise. RPE can be used as a general assessment of effort because it attempts to integrate physiological (exercise intensity, etc.) and psychological assessments. Therefore, in addition to the same exercise intensity, the physiological and psychological stress caused by high temperature and humidity may be the cause of the above results^83,84^. Moreover, numerous research investigations have shown that subjects with higher SR usually exhibit higher RPE^85^, and our results showed no significant difference in SR between the 4 groups, which explains the lack of significant difference between the 4 groups. Inconsistent with the present study, previous studies have shown a significant increase in SR at humidity levels greater than 60%^19,86^_._

Finally, we would like to point out that this study uniformly used 24 degrees Celsius, 40 RH during the recovery process. In actual training, after completing 4 × 4 HIIT training, college football players will enter the lounge (thermoneutral environment) for rest. One study have shown that compared to high temperature and humidity environments, thermoneutral environment do not cause significant differences in HR and lactate levels at baseline and recovery stages^46^. Therefore, a thermoneutral environment as a recovery condition will not result in differences in HR and lactate levels, and this may not result in differences in athletic ability during the recovery period. In addition, studies have shown that different temperature and humidity conditions do not have a significant impact on the autonomic nervous system^55^ and the MAP^59^. Therefore, the above recovery conditions may not affect the results of HRV and MAP. High temperature and humidity environments can lead to a decrease in thermal conductivity efficiency^87^. Therefore, an increase in conductivity efficiency may be the reason why there is no significant difference between T_T_ and T_S_ under thermoneutral environment. In addition, thermoneutral environment do not cause differences in TS and the RPE^88^, so there is no significant difference except for a short-term difference when leaving a high temperature and humidity room.

## Conclusion and suggestions

The physiological effects caused by hot and humid environments were generally greater compared to normal environments but had no effect on the athletic performance of college football players, and the above reasons may be caused by the intensity and short duration of the exercise protocol. This study was conducted under laboratory conditions. If conditions permit, it is recommended to experiment with natural environmental conditions. Performing HIIT exercises in a high temperature and humidity environment and resting in a normal environment is a good means of recovery.

### Limitations

Due to the need to strictly control the ambient temperature and humidity of the subjects and to ensure their safety, the cycle ergometer used in this experiment may not be as compatible with the soccer player's movement mode as using treadmill as an exercise device. Furthermore, the subjects of this experiment were college soccer players not representative of the physiological responses and performance of elite soccer players.

## Data Availability

The datasets used and/or analyzed during the current study are available from the corresponding author on reasonable request.
